# Integration of Methane Steam Reforming and Water Gas Shift Reaction in a Pd/Au/Pd-Based Catalytic Membrane Reactor for Process Intensification

**DOI:** 10.3390/membranes6030044

**Published:** 2016-09-19

**Authors:** Bernardo Castro-Dominguez, Ivan P. Mardilovich, Liang-Chih Ma, Rui Ma, Anthony G. Dixon, Nikolaos K. Kazantzis, Yi Hua Ma

**Affiliations:** Center of Inorganic Membrane Studies, Department of Chemical Engineering, Worcester Polytechnic Institute, 100 Institute Road, Worcester, MA 01609, USA; ivanpm@wpi.edu (I.P.M.); lma@wpi.edu (L.-C.M.); rma2@wpi.edu (R.M.); agdixon@wpi.edu (A.G.D.); nikolas@wpi.edu (N.K.K.); yhma@wpi.edu (Y.H.M.)

**Keywords:** hydrogen production, catalytic membrane reactor, methane steam reforming, water-gas-shift reaction, reaction coupling

## Abstract

Palladium-based catalytic membrane reactors (CMRs) effectively remove H_2_ to induce higher conversions in methane steam reforming (MSR) and water-gas-shift reactions (WGS). Within such a context, this work evaluates the technical performance of a novel CMR, which utilizes two catalysts in series, rather than one. In the process system under consideration, the first catalyst, confined within the shell side of the reactor, reforms methane with water yielding H_2_, CO and CO_2_. After reforming is completed, a second catalyst, positioned in series, reacts with CO and water through the WGS reaction yielding pure H_2_O, CO_2_ and H_2_. A tubular composite asymmetric Pd/Au/Pd membrane is situated throughout the reactor to continuously remove the produced H_2_ and induce higher methane and CO conversions while yielding ultrapure H_2_ and compressed CO_2_ ready for dehydration. Experimental results involving (i) a conventional packed bed reactor packed (PBR) for MSR, (ii) a PBR with five layers of two catalysts in series and (iii) a CMR with two layers of two catalysts in series are comparatively assessed and thoroughly characterized. Furthermore, a comprehensive 2D computational fluid dynamics (CFD) model was developed to explore further the features of the proposed configuration. The reaction was studied at different process intensification-relevant conditions, such as space velocities, temperatures, pressures and initial feed gas composition. Finally, it is demonstrated that the above CMR module, which was operated for 600 h, displays quite high H_2_ permeance and purity, high CH_4_ conversion levels and reduced CO yields.

## 1. Introduction

Methane steam reforming (MSR) is a well-established production method that currently generates 95% of hydrogen in the U.S. [1]. Conventionally, this reaction is carried out at high temperatures (700–1000 °C) and mild pressures (3–25 bar), which results in the production of CO and H_2_ with little CO_2_ as a byproduct. Carbon monoxide is reacted downstream in two, high- and low-temperature, water-gas-shift (WGS) reactors to further generate H_2_ and CO_2_. Equations (1)–(3) [2] show both chemical reactions where MSR is highly endothermic while WGS is exothermic. After the reaction, the purification of H_2_ is traditionally carried out in a pressure swing adsorption (PSA) unit. Notice that in the conventional process, both MSR and WGS reactions are limited by their thermodynamic equilibrium when carried out in customary packed bed reactors (PBRs).

(1) $$\text{MSR I }CH_{4}+H_{2}O↔CO+3H_{2}\;\Delta {\text{H}}_{298\text{K}}=206\text{ kJ}/\text{mol}$$

(2) $$\text{WGS }CO+H_{2}O↔CO_{2}+H_{2}\;\Delta {\text{H}}_{298\text{K}}=-41\text{ kJ}/\text{mol}$$

(3) $$\text{MSR II }CH_{4}+2H_{2}O↔CO_{2}+4H_{2}\;\Delta {\text{H}}_{298\text{K}}=165\text{ kJ}/\text{mol}$$

The application of membrane technology is economically attractive since it helps to reduce the number and size of equipment units needed, while simultaneously enhancing chemical conversions. The higher efficiency of the so-called catalytic membrane reactors (CMRs) results from the constant product removal from the reaction zone, through a selective membrane, altering the composition of the system. This change in composition allows accomplishing higher conversions, and it is often referred to as process intensification. Specifically, H_2_-selective membranes can improve MSR and WGS conversions while generating more hydrogen. Palladium-based membranes have been shown to be the best candidates for implementation in these processes since they can isolate hydrogen from gas mixtures in large quantities and at high purities while maintaining high stability and robustness. Ma et al. [3,4] have demonstrated the performance of different pilot-scale Pd and Pd-alloy membranes under industrial conditions. These membranes were capable of producing 1.16 kg/day of H_2_ with a purity of 99.89% under actual coal-derived syngas at 450 °C. Although a reduced H_2_ permeance was displayed due to the presence of contaminants, the stability, robustness and physical integrity of the membranes were successfully demonstrated.

In the case of CMR technology, the literature has shown membrane application up to a pilot-scale magnitude [5], as shown in Table 1. Palladium membranes are capable of operating at temperatures between 300 and 600 °C, matching the temperatures of high-temperature WGS catalysts (310–450 °C). A low-temperature WGS unit is no longer needed, since high CO conversions can be achieved through a single CMR unit. Furthermore, the effect of process intensification in MSR allows operating this process at mild temperatures (Table 1) while maintaining high conversions, preventing coking and reducing the CO yield. The experimental tests shown in the literature demonstrate the effect of process intensification; nevertheless, the scale of operation is relevant for the industrial deployment of this technology. Few pilot-scale tests have been published in this field, including the work presented by Catalano et al. [6], where 5.6 m^3^ of H_2_ per day were produced through a WGS membrane reactor at 440 °C and 20 bar. Furthermore, Patrascu et al. [7] showed the performance of methane steam reforming (MSR) in a large-scale CMR achieving a permeate flux of 1.6 NL/min at a temperature of 525 °C and a pressure of 10 bar and utilizing a membrane with a surface area of 175 cm^2^. Furthermore, economically, CMR technology has shown success when integrated in power and hydrogen production plants. Kazantzis et al. reported the specific market and regulatory conditions under which Pd-based CMRs can be successfully integrated in the pertinent energy systems [8,9].

This work aims to implement the integration of one more processes into a single unit. Specifically, the operation of a large-scale Pd-based CMR module is shown where both MSR and WGS reactions are integrated in a single unit, in order to provide high CH_4_ yields with little or no CO. In the CMR, the first catalyst (MSR) is confined on the shell side of the reactor, while the WGS catalyst is positioned subsequently in series. A tubular Pd/Au/Pd membrane situated throughout the reactor continuously removes the H_2_ to yield higher conversions. The higher shell pressure facilitates the acquisition of clean pressurized CO_2_ and water, while ultrapure H_2_ is obtained in the permeate stream. Furthermore, for comparison, the reaction is carried out in a conventional packed bed reactor for both configurations: MSR and multistage MSR-WGS (five layers). Additionally, a two-dimensional computational fluid dynamics (CFD) model was developed in order to further analyze the properties of this unit. Please notice that the aim of this work is a proof-of-concept approach that explores the potential use of catalysts packed in series and/or in parallel to enhance conventional processes.

This work is structured as follows: Section 2 presents the methodological framework, as well as an explicit description of the experimental procedure and CFD development. Section 3 encompasses the results associated with the conventional reactors and the CMR’s performance characteristics along with the pertinent simulation results accompanied by a thorough discussion related to the effect of coupling two catalysts in one unit. Finally, some concluding remarks are offered in Section 4.

## 2. Methodology

### 2.1. Membrane Fabrication

A composite Pd/Au/Pd membrane was prepared on a 1.27-cm OD and 38.1-cm in length 316-L porous stainless steel (PSS) support with media grade of 0.5 μm. The total permeable area of the membrane was 152 cm^2^. One end of the membrane was welded to a 316-L nonporous capped tube while the other end was welded to a nonporous tube. The support showed an initial He flux of 200 L/min at a pressure difference of one bar. To synthesize the membrane, the support was first covered with sol-gel and then calcined at 600 °C for 12 h. After calcination, the supports were graded following a previously-reported procedure with medium and fine pre-activated powder, provided by Johnson Matthey (Royston, UK), based on a 2 wt % Pd-alumina catalyst without any additional activation or treatment [23,24,25]. Notice that the grading procedure reduced the He leak across the membrane by 3 orders of magnitude, as shown in Table 2. After grading, the surface of the membrane was activated with SnCl_2_-PdCl_2_, and then, electroless plating was used to deposit a dense Pd layer. A thin gold layer of 0.2 μm was deposited on top of the palladium surface via conventional electroplating. Notice that gold has been shown to enhance the properties of Pd-based membranes, such as permeance, stability and contaminant-recoverability [26], and therefore, was used in this work. Finally, to provide active sides on this asymmetric membrane and further reduce the He leak present, a pure Pd topmost layer was deposited. The thickness of the membrane was estimated by gravimetric methods. The final composition and leak of the membrane was 6.9 Pd/0.2 Au/3.2 Pd and <0.01 sccm/bar, respectively. The thicknesses and He leak at each step of the synthesis are shown in Table 2.

### 2.2. Reaction Tests and Membrane Characterization

The H_2_ permeation tests and reactions were performed in the same WGS-CMR rig previously reported by Catalano et al. [6]. The composition of the feed was controlled by mass flow controllers and premixed with steam generated in a preheater. The wet mixture was fed to the reactor, which contains the membrane surrounded by the catalysts. The catalysts used for MSR and WGS were a nickel-based catalyst (HiFUEL R110, Alfa-Aesar, Lancashire, UK) and an iron-chrome catalyst (HiFUEL W210, Alfa-Aesar), respectively; these catalysts were crushed and sieved (16/+40 mesh) before usage. The water of the retentate was condensed, while the product and retentate flow rates were passed through water absorbent beds before the composition was measured by mass flow meters and a gas chromatograph [6]. Three main experiments were conducted on the CMR-rig, including: (i) MSR in a conventional packed bed reactor (PBR); (ii) multi-staged (5 layers) MSR/WGS in a PBR; and (iii) MSR/WGS in a CMR. It is important to note that no sweep gas was utilized in any of the experiments presented in this work.

**Figure 1:**
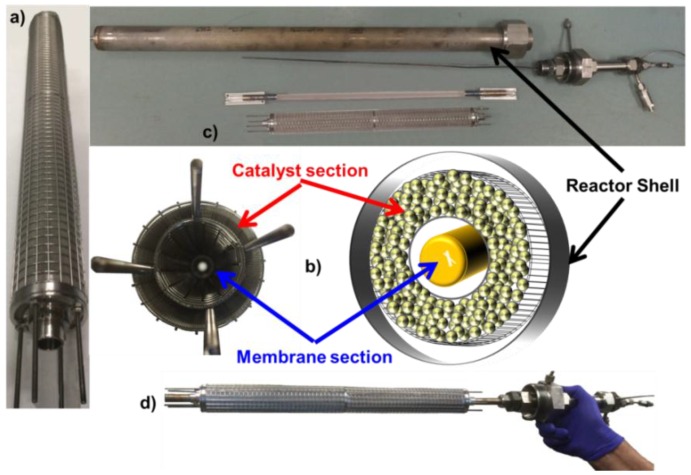
Cage, catalyst and membrane configurations used in this work: (**a**) assembled protective cage without a membrane; (**b**) cross-sectional view of the cage and its schematic representation; (**c**) different components of the catalytic membrane reactor (CMR) module; (**d**) integrated cage-catalyst-membrane

For the CMR reaction, a protective cage was designed in order to prevent any potential damage of the membrane caused by the friction of the catalyst particles and the wall of the membrane, as previously reported in the literature [22,27]. The cage was made out of stainless steel grids, and it consisted of two concentric confines; one surrounded the membrane, while the other was used to hold the catalyst in place, as shown in Figure 1b. Notice that the surface of the membrane was never in contact with the grid of the cage. The catalyst section of the cage had a volume of 480 cm^3^, and it was filled with 120 g of MSR catalyst and 120 g of WGS catalyst for the membrane reactor, while the PBR was packed in 5 sections. This membrane-catalyst cage system can be up-scaled in order to develop multi-tube CMR modules. 

### 2.3. Mathematical Modeling Framework

**Figure 2:**
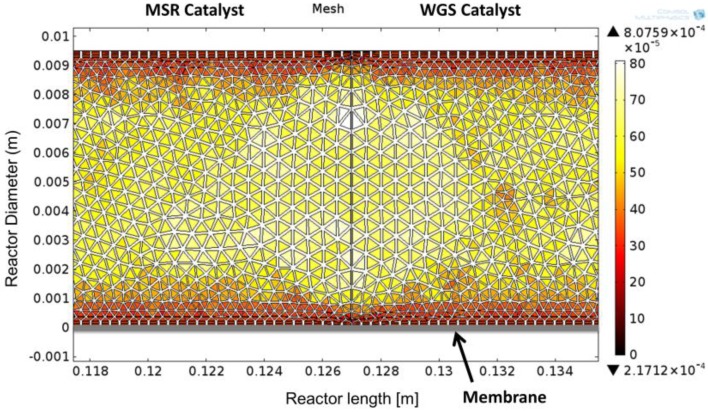
Configuration of the CMR simulation depicting the two catalyst sections, the location of the membrane and the size and geometry of the used mesh.

A detailed modeling framework, helpful for the analysis of CMRs, has been used for the interpretation of data [5], and accordingly, a 2D computational fluid dynamics (CFD) model was developed in COMSOL Multiphysics 4.3b (COMSOL, Inc., Burlington, VT, USA) in order to examine the properties of the module and compare theoretical values with experimental results. A 2D configuration of the model was chosen in order to include the non-ideal flow effects that occur in the reactor due to the axisymmetry of the reactors. The performance of the simulation was compared against the experimental values obtained in this work and, for MSR, against the 1D model presented by Ayturk et al. [2], where a 99.4% accuracy was found when compared to other literature sources, including conventional PBRs and CMRs. Figure 2 shows the configuration of the 2D model, where the MSR catalytic section is located adjacent to the feed flow stream, followed by the WGS section upstream; the membrane was specified to be at the bottom, taking advantage of the symmetrical configuration of the reactor module; additionally, the mathematical mesh, displayed in Figure 2, is used to solve the momentum and continuity equations for the retentate side (Equations (4)–(6)) (COMSOL Multiphysics). The Darcy–Forchheimer law was applied in the present model accompanied by the following assumptions:

(1) Isothermal conditions;
(2) Steady state;
(3) Laminar flow;
(4) Non-slip boundary condition for the fluid flow;
(5) Negligible effect of the protective cage on the flow pattern.

The modified Navier–Stokes equation for a fixed bed porous medium is:

(4) $$\frac{\rho }{\varepsilon_{p}}\left(\left(u\cdot \nabla \right)\frac{u}{\varepsilon_{p}}\right)=\nabla \cdot \left[-\mathrm{pI}+\frac{\mu }{\varepsilon_{p}}\left(\nabla u+{\left(\nabla u\right)}^{T}\right)-\frac{2u}{3\varepsilon_{p}}\left(\nabla \cdot u\right)I\right]-\left({\mu k}^{-1}+\beta_{F}\left|u\right|\right)u+F$$

where $\varepsilon_{p}$ represents the system porosity and $\beta_{F}$ is the Forchheimer coefficient:

(5) ∇·(ρu)=0

(6) $$\nabla \cdot \left(-D_{i}\nabla c_{i}\right)+u\cdot \nabla c_{i}=R_{i}$$

The reaction rates for MSR were specified as [2]:

(7) $$r_{1}=\;\frac{k_{1}}{P_{H2}^{2.5}}\times \frac{P_{\text{CH}4}P_{H2O}-\left(P_{H2}^{3}P_{\mathrm{CO}}/K_{1}\right)}{{\mathrm{DEN}}^{2}}$$

(8) $$r_{2}=\;\frac{k_{2}}{P_{H2}}\times \frac{P_{\mathrm{CO}}P_{H2O}-\left(P_{H2}P_{\text{CO}2}/K_{2}\right)}{{\mathrm{DEN}}^{2}}$$

(9) $$r_{3}=\;\frac{k_{3}}{P_{H2}^{3.5}}\times \frac{P_{\mathrm{CH}4}P_{H2O}^{2}-\left(P_{H2}^{4}P_{\mathrm{CO}}/K_{3}\right)}{{\mathrm{DEN}}^{2}}$$

where DEN is defined as:

(10) $$\mathrm{DEN}=1+K_{\mathrm{CO}}P_{\mathrm{CO}}+K_{H2}P_{H2}+K_{\text{CH}4}P_{\text{CH}4}+\;\frac{K_{H2O}P_{H2O}}{P_{H2}}$$

Notice that r_1_, r_2_ and r_3_, correspond to the reactions specified in Equations (1)–(3), respectively. Refer to the previous literature for the kinetic, adsorption and equilibrium constants [2]. Furthermore, the reaction rate used for the WGS reactor model over Fe-Cr-based catalyst was specified as [28]:

(11) $$r_{4}=\;10^{2.845}\cdot e^{\frac{-111}{\mathrm{Rg}\cdot T}}\cdot P_{\mathrm{CO}}^{1.0}P_{\mathrm{CO}2}^{-0.36}P_{H2}^{-0.09}\cdot \left[1-\frac{P_{H2}P_{\mathrm{CO}2}}{{K\;P}_{H2O}P_{\mathrm{CO}}}\right]$$

where K is the equilibrium constant.

The reaction model is homogenous as the internal effectiveness factor was calculated to be 1. The internal effectiveness factor is defined as the actual rate of reaction divided by the rate of reaction that would occur if the entire internal surface of the catalytic particle would be exposed to the external conditions.

Additionally, the flux across the membrane ($N_{i}$) was based on Sieverts’ law as follows [2,29]:

(12) $$N_{i}=\;\bar{P_{H2}}\left(\sqrt{P_{H2}^{\mathrm{Shell}}}-\sqrt{P_{H2}^{\mathrm{Tube}}}\right)$$

where $P_{H2}^{\mathrm{Shell}}\;$ and $P_{H2}^{\mathrm{Tube}}\;$represent the hydrogen partial pressure at the retentate side and the permeate side, respectively, and $\bar{P_{H2}}$ is the permeance of the membrane obtained experimentally. Furthermore, the calculation of binary fluid diffusion coefficients ($D_{m}$) was estimated according to standard engineering procedures [30]:

(13) $$D_{m}=\;0.0018583\sqrt{T^{3}\left(\frac{1}{M_{A}}+\frac{1}{M_{B}}\right)}\frac{1}{{P\sigma }_{\mathrm{AB}}^{2}\varphi_{D,\mathrm{AB}}}$$

where $M_{i}$ is the molecular weight of component i, P is the pressure of the system, $\sigma_{\mathrm{AB}}^{2}$ denotes the parameters of the Lennard–Jones potential between molecules A and B and $\varphi_{D,\mathrm{AB}}$ represents the collision integral for diffusion.

The longitudinal and transversal dispersion D_L_ and D_T_ are calculated using the equations below [31]:

(14) $${\mathrm{Pe}}_{m}=\frac{d\cdot u}{D_{m}}$$

(15) $$1/{\mathrm{Pe}}_{L}\;=1/\left(\tau \cdot {\mathrm{Pe}}_{m}\right)+1/2$$

(16) $$1/{\mathrm{Pe}}_{T}\;=1/\left(\tau \cdot {\mathrm{Pe}}_{m}\right)\;+1/12$$

(17) $$D_{L}=u\cdot d/{\mathrm{Pe}}_{L}$$

(18) $$D_{T}=u\cdot d/{\mathrm{Pe}}_{T}$$

where ${\mathrm{Pe}}_{m},\;{\mathrm{Pe}}_{L}$ and ${\mathrm{Pe}}_{T}$ are the molecular Péclet number, the longitudinal Péclet number and the transversal Péclet number, respectively; τ denotes tortuosity, and d represents the catalyst particle diameter.

The conversion of methane was defined as [2]:

(19) $$X_{\text{conversion}}=\;\frac{F_{\text{CH}4,\mathrm{feed}}-F_{\text{CH}4,\mathrm{ret}}}{F_{\text{CH}4,\mathrm{feed}}}$$

## 3. Results and Discussion

### 3.1. He Leak Tests and H_2_ Permeation Tests of the Membrane

**Figure 3:**
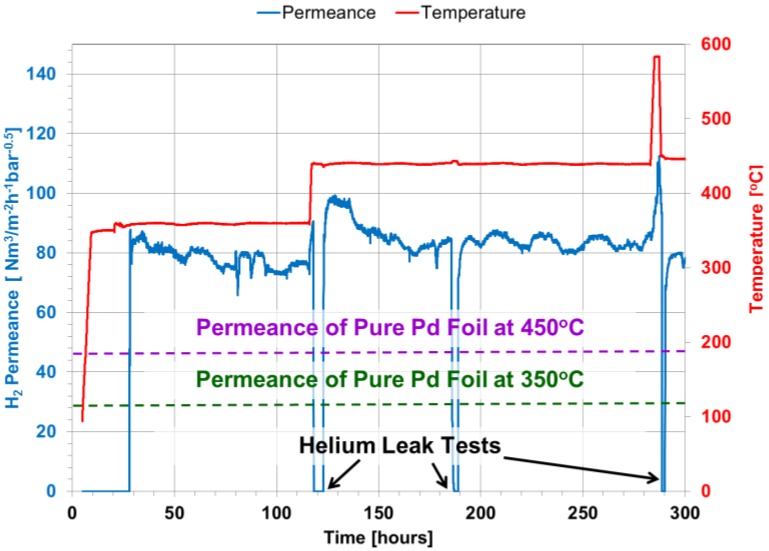
Hydrogen permeance, He leak tests at 350, 450 and activation at 585 °C at different elapsed times.

After the module was installed in the CMR rig, the temperature of the membrane module was increased from room temperature to 350 °C under He gas at a rate of 1 °C/min and a pressure of 2 bar. At this temperature, a helium leak test showed an undetectable leak, and H_2_ was introduced to the module. Hydrogen permeance was measured as a function of time continuously every 30 s as shown in Figure 3. After 80 h, the temperature was increased to 450 °C, displaying a slight increase in H_2_ permeance. The temperature was kept for 160 h, and two helium leak tests were performed displaying undetectable He leak. Notice that on the first He leak test, steam was fed to the system along with He for one hour to fully oxidize the WGS catalyst. The membrane showed a H_2_ permeance of 70 and 80 Nm^3^·m^−2^·h^−1^·bar^−0.5^ at 350 and 450 °C, respectively. After 290 h of continuous testing, the module temperature was increased to ~600 °C under a pure H_2_ stream for 3 h to activate the MSR catalyst. Notice that after activation, the membrane presented a He leak of 0.4 sccm/bar at 450 °C. The asymmetric Pd/Au/Pd membrane showed high H_2_ flux and an ideal H_2_/He selectivity of over 4300 after the catalyst was activated. Even though it has been shown that to improve the thermal stability of the membranes, porous Hastelloy and Inconel supports perform better than PSS at temperatures higher than 500 °C [32], the membrane showed a high thermal stability.

Moreover, Gade et al. [33] showed that unannealed Pd-Au membranes require ~300 h under typical operating conditions to fully anneal in situ the Pd-Au surface of the membrane and consequently reach a steady H_2_ flux. Nevertheless, as shown in Figure 3, after H_2_ feed was introduced into the system at 350 °C, the H_2_ flux across the membrane reached a steady state very quickly. This effect could be the result of plating Pd on top of the Au surface, which added active sites for the permeance to occur.

Pure Pd membrane foils have shown a H_2_ permeance that follows the Arrhenius correlation, as shown in Equation (20), where *t* is the thickness of the membrane in μm, 15,630 is the activation energy in J/mol and 6322.7 is the pre-exponential factor in m^3^·μm·m^−2^·h^−1^·atm^−0.5^ [2]. Furthermore, considering that the presented Pd/Au/Pd membrane has a Pd layer of 10.1 μm, the expected permeance of its pure Pd foil analog is 47 Nm^3^·m^−2^·h^−1^·bar^−0.5^ at 450 °C. It is important to mention that the hydrogen permeance of the presented Pd/Au/Pd membrane is superior by a factor of 1.7. This enhanced behavior of the membrane is due to the presence of gold, which as previously reported [3,34] can raise the permeance up to two-times higher due to an increase in diffusivity. Although the amount of gold in the presented membrane is 2%, which is below the optimum 5% [35], the membrane displayed an excellent and stable H_2_ flux.

(20) PH2¯=[6322.7 e−15630RT]t

### 3.2. MSR in a Conventional Packed Bed Reactor: Single Catalyst

**Figure 4:**
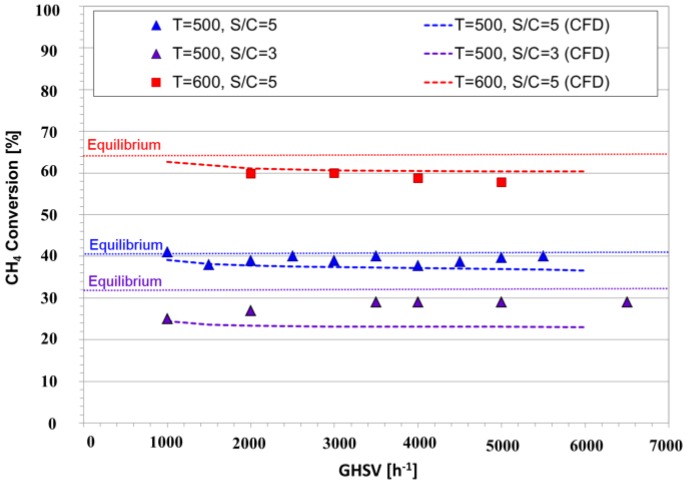
Experimental and computer simulation methane conversions in a packed bed reactor (PBR) as a function of different space velocities and conditions at a total pressure of 2 bar.

Methane steam reforming was carried out in a conventional packed bed reactor (PBR) to experimentally demonstrate the effect of process intensification and the presence of the secondary catalyst. As mentioned before, the major advantage of the CMR compared to a conventional PBR is the conversion enhancement of the equilibrium-limited MSR by removing in situ the produced H_2_. Therefore, in order to study the performance of a PBR, a solid stainless steel pipe was placed instead of the membrane in order to maintain identical geometric features of the CMR reactor. As shown in Figure 4, different space velocities, temperatures and steam-to-carbon ratios were used to investigate the performance of the PBR; all reaction conditions were set to a total pressure of 2 bar, since higher pressures did not show significant changes in the reaction performance. Notice that the catalyst loading was arranged in such a way that the operating GHSV was of 5000 h^−1^ as specified by the provider of the catalyst. Furthermore, the experimental results were graphically depicted along with the computational simulation outcomes as shown in Figure 4.

We examine the performance of the PBR by analyzing its methane conversion at 500 °C and a steam-to-carbon ratio of three. It is observed that even at small space velocities, methane conversion is below its chemical equilibrium (shown as a dotted line in Figure 4); this effect is caused by the reduction of the contact time of methane with the catalyst. Furthermore, the effect of temperature on the conversion of methane is clear; it increased from 40% to 60% when the temperature of the reactor was increased from 500 to 600 °C. This is in agreement with the fact that MSR is an endothermic reaction, which is highly favored by high temperatures.

Additionally, adding steam has a positive effect on the reaction, doubling the conversion when the steam-to-carbon ratio is increased from 3 to 5. Furthermore, excess steam is generally present in the MSR process, since it not only increases conversion, but also prevents coke formation. It is important to mention that the results presented in this work are similar to the results reported in the pertinent literature [2,36]. Additionally, the CFD simulation results, shown in Figure 4, match the experimental data with an average error of 7.8%. The experiments were carried out in a pilot-scale module, and therefore, these results were more susceptible to divergence from controlled settings.

### 3.3. MSR/WGS in a Conventional Packed Bed Reactor: Dual Catalyst

**Figure 5:**
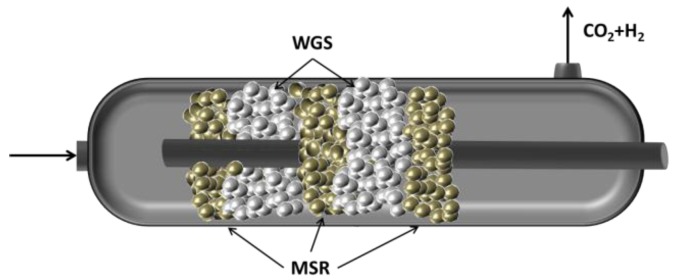
Conceptual illustration of the catalyst distribution throughout the PBR reactor with the left side cut away to show the tube and the catalyst.

The reforming of methane and the water gas shift reactions were studied in a conventional packed bed reactor (PBR) to demonstrate the effect of the secondary catalyst and thus effectively demonstrate the presence of a membrane. The reactor was packed in stages while a solid stainless steel pipe was used instead of a membrane in order to maintain the geometry of the module, as shown in Figure 5. The PBR was packed in series with a fresh Ni-based reforming catalyst and a Fe-Cr-based WGS catalyst with an overall proportion of 20% and 80% for reforming and WGS, respectively. The configuration of the catalysts within the reactor, shown in Figure 5, was split as MSR-WGS-MSR-WGS-MSR. The MSR-WGS reactor was tested at 475 °C since the Fe-Cr catalyst temperature limit is specified by the provider to be of 500 °C. After packing the module, steam and He were fed to the system to oxidize the WGS catalyst. The catalyst emitted H_2_, and therefore, oxidation continued until H_2_ was not detectable by the GC [6]; this process took around 1 h. Afterwards, the temperature of the module was increased to ~600 °C under pure H_2_ stream for 3 h to activate the MSR catalyst. After these procedures, the reaction tests were carried out. Notice that a CFD simulation for this multistage packing configuration was performed to further analyze the PBR.

**Figure 6:**
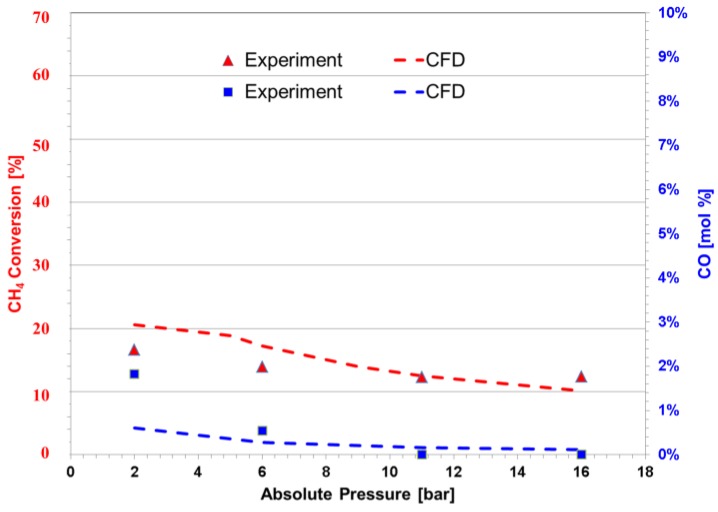
Experimental and computational simulation of two catalysts in a PBR as a function of different pressures at a GHSV of 3500 h^−1^, a temperature of 475 °C and a steam-to-carbon ratio of five.

The conversion of methane at 475 °C, 2 bar and a GHSV of 3500 h^−1^ was found to be 18%, as shown in Figure 6; however, it decreased slightly as the pressure was increased. It is important to mention that the purpose of adding the WGS catalyst is to prevent or reduce the formation of CO in the module. As shown by Figure 6, it can be observed that although in small quantities, CO is present in the product of the reaction. For both experiments and simulations, the amount of CO reduces as pressure increases; this indicates that the production of CO may be hindered by pressure or that the activity of the WGS catalyst is favored at higher pressures (Equation (2)). Given the stoichiometry of MSR (Equations (1)–(3)), a reduction of methane conversion and simultaneously CO generation as the pressure of the reactor increases is expected; at the same time, as reported by Atwood et al. [37], the WGS reaction intensifies at higher pressures. These two mechanisms contribute to obtaining lower CO yields.

**Figure 7:**
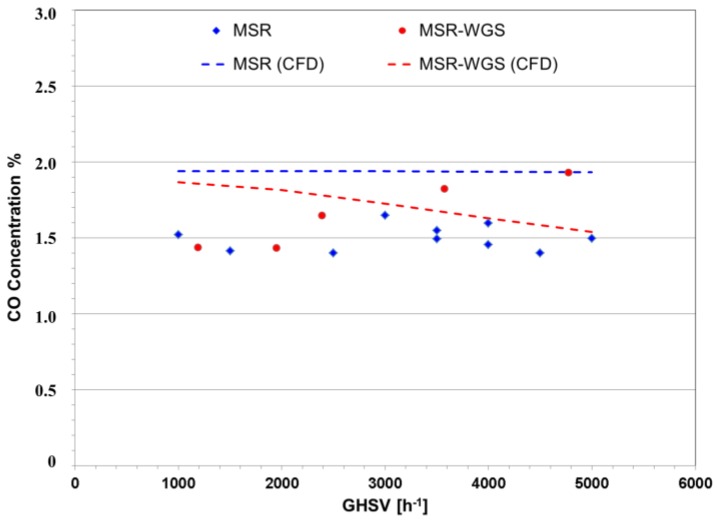
Concentration of CO in conventional PBRs containing a single MSR catalyst and a dual MSR-WGS catalyst at a pressure of 2 bar, a temperature of 475 °C and a steam-to-carbon ratio of five.

**Figure 8:**
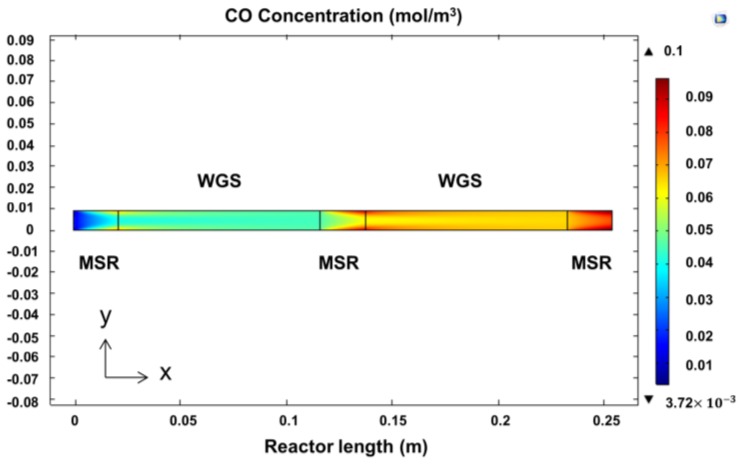
CFD concentration profile of CO inside in a PBR containing MSR-WGS catalysts.

It is important to notice that in this dual-catalyst reactor, the WGS reaction occurs in the presence of a significant amount of H_2_, which limits its performance. Figure 7 shows that even though lower theoretical CO is present in the dual-catalyst reactor, the experimental results of the pilot-scale bed appeared to be hindered by the intrinsic error in the measurements. Nevertheless, through the simulation, it is found that as the space velocity increases, the difference in CO production decreases further for the dual-catalyst bed. This effect can be attributed to two factors: (i) the reduced presence of H_2_; and (ii) lower concentrations of CO. The aforementioned factors are generated due to reduced CH_4_ conversions. Additionally, a surface plot of CO concentration through the reactor module is shown in Figure 8 to illustrate the effect of the water-gas-shift catalyst. At first, CO is generated on the first MSR catalyst bed section, followed by its consumption by the WGS reaction zone. The next MSR layer induces the production of more CO, which is later reduced by the following WGS segment. Finally, the MSR catalyst at the end of the PBR increases the overall CO concentration inside the reactor.

### 3.4. MSR/WGS in a Catalytic Membrane Reactor

**Figure 9:**
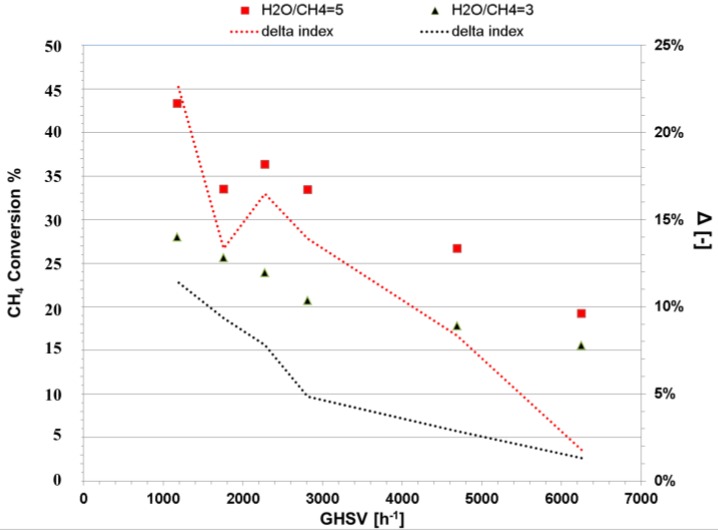
Experimental methane conversion of the dual catalyst CMR represented as scatter points and the difference between CMR and PBR at 5 bar, at a temperature of 475 °C.

The CMR was packed with two layers of catalysts in series only (in contrast with the five layers in the PBR) and with a membrane placed at the center of the reactor to remove in situ the H_2_ generated by the reactions. The experimental CH_4_ conversion results are shown in Figure 9 for different steam-to-carbon ratios, a temperature of 475 °C and a pressure of 5 bar. Notice that, in contrast with conventional PBRs, the pressure has a significant effect on the effectiveness of CMR technology. Since the rate of removal of H_2_ is a function of its partial pressure, higher pressures will ensure a better performance. Furthermore, the CMR in this reactor was not tested at 2 bar (as the PBR); because at such a low pressure difference, it is expected to observe reverse flow (from the permeate side to the reacting side) since H_2_ is pure on the permeate side. The highest conversion achieved was of 43.3% at a steam-to-carbon ratio of five and a space velocity of 1172 h^−1^. Furthermore, it is found that as the GHSV was increased, the conversion of methane decreased accordingly; this was caused by the reduction of residence time in the reactor. In addition, the amount of water influenced the reaction significantly; a steam-to-carbon ratio of five produced about 20% higher CH_4_ conversion than a ratio of three. Notice that the H_2_ purity generated by this Pd/Au/Pd membrane was 99.94% throughout a testing time of 350 h under MSR/WGS conditions. The best flux achieved by the CMR under optimum conditions was over 500 NL/day. After the experiments were terminated, the surface of the membrane did not show carbon deposition for the reason that the protective cage separated effectively the reaction zone from the membrane.

The reactor performance indicator for process intensification was quantitatively analyzed based on the Δ-index previously reported by Ayturk et al. [2]. This index is represented in Equation (21) as the difference between the CH_4_ conversion achieved by the CMR and the one by PBR under similar conditions.

(21) $$\Delta \;=\;X_{CH4}^{CMR}-\;X_{CH4}^{PBR}$$

It is important to mention that the PBR was not operated experimentally at 475 °C and 5 bar; consequently, the CFD performance outcome of the conventional PBR was utilized to estimate the Δ-index of this work. The Δ-index represented in Figure 9 shows that at all GHSV, the conversion of methane increases when sized against a conventional reactor. Nevertheless, the concept of process intensification is better appreciated at low space velocities since H_2_ has a better rate of removal and the contact time of the gases with the catalysts increases.

**Figure 10:**
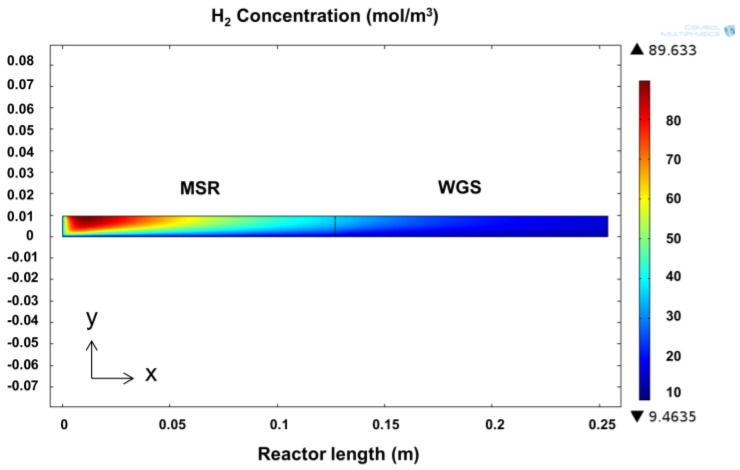
Concentration profile of H_2_ inside in a dual MSR-WGS catalytic membrane reactor.

For the simulation result, as expected, compared to a PBR, the constant removal of H_2_, shown by the hydrogen concentration profiles in Figure 10, changes the composition of the retentate, allowing both reactions to proceed further. In Figure 10, it is possible to observe that as soon as the feed stream (on the left) is in contact with the catalyst bed, H_2_ is generated and increases as the reaction proceeds; this continues until the H_2_ partial pressure in the retentate is high enough to provide the driving force for the membrane to start removing it. Notice that even though the reaction continues to take place in the module, an increase in H_2_ concentration is no longer observed; this effect is caused by the rate of removal overcoming the rate of reaction. Furthermore, it is possible to observe, from top to bottom, a gradual reduction in H_2_ concentration caused by the presence of the membrane. This reduction in H_2_ concentration adjacent to the surface of the membrane causes a H_2_ depleted boundary layer formed by low radial diffusion rates. This effect is often referred to as concentration polarization, and it can significantly reduce the performance of the membrane [38].

To further characterize the performance of the membrane reactor, the product of Damkohler and Péclet numbers (DaPe number) is utilized, since it provides the ratio of maximum reaction rate over the maximum permeation rate per volume [39]. In PBRs, the Damkohler number (Da) exemplifies the performance of the reactors, since it shows the ratio of the reaction rate over the convective mass transport of the reactant; while in membrane technology, the Péclet number shows the relative convection transport rate over the diffusive rate (permeation). Consequently, the product DaPe dictates the overall effectiveness of the CMR; for instance, having a DaPe > 1 means that the permeation rate is low, and thus, the H_2_ rate of removal through the membrane is the limiting factor of the reactor’s productivity. As reported by Battersby et al. [39], the DaPe number can be estimated as shown in Equation (22) where X_equilib_ is the conversion achieved when the reaction is thermodynamically at equilibrium, and X_actual_ is the conversion displayed by the membrane reactor. Most of the DaPe numbers displayed by this CMR, shown in Table 3, are lower than one; this implies that the rate of H_2_ removal is high enough to change the pseudo-equilibrium state favorably to achieve higher conversions. Notice that the term “pseudo-equilibrium” is used to describe the situation where the reaction product (H_2_) is independently manipulated, by the use of a permeable membrane [39]. Furthermore, Table 3 shows that at high GHSV, the DaPe number approaches one, implying that the rate of reaction matches the maximum permeation equivalent. It is important to mention that it is considered that the optimum design of a CMR should operate at a theoretical DaPe = 1.

(22) $$DaPe\;=\;\frac{X_{equilib}}{X_{actual}}$$

**Figure 11:**
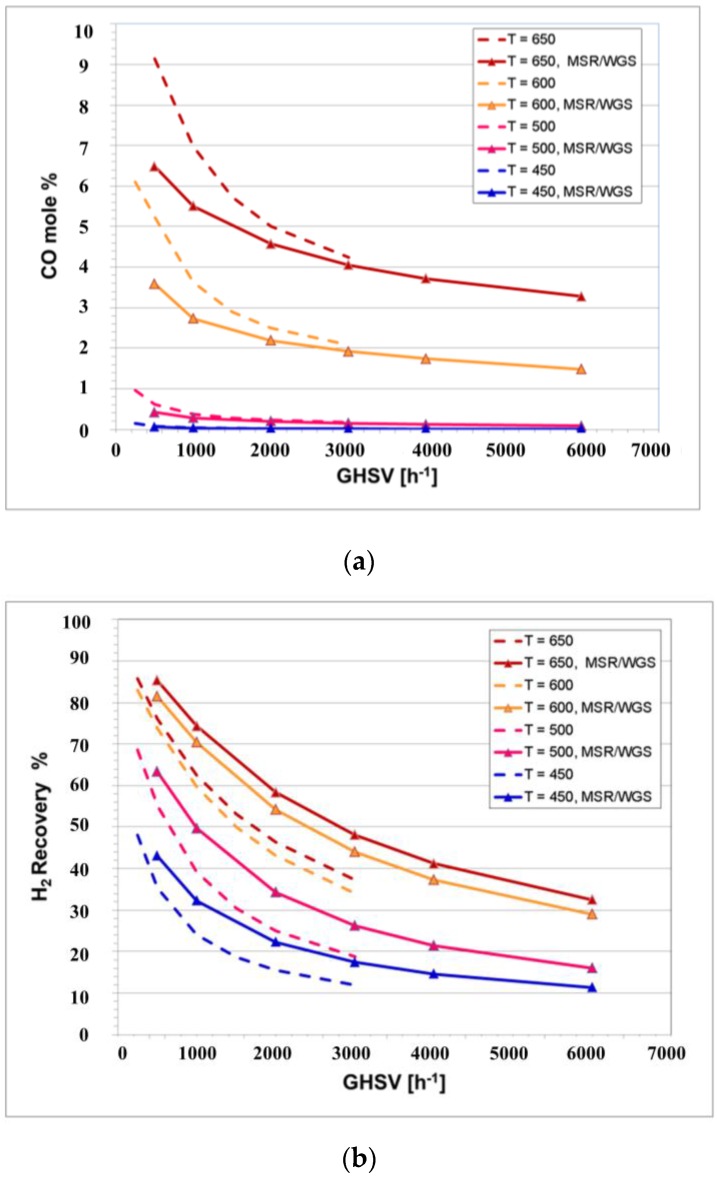
(**a**) CO yield and (**b**) H_2_ recovery for single and dual CMRs at 5 bar and a carbon-to-steam ratio of five.

The concentration of CO in the system was undetectable in this dual catalytic CMR. However, it is not clear if this is the result of the presence of the secondary WGS catalyst or if it is caused by the presence of a H_2_-permeable membrane, as previously reported [6,12,18,19]. For instance, Lin et al. described a reduction of CO yield from 50% down to <2% in a Pd-based CMR [12]. Therefore, to observe the effect of the secondary catalyst, a simulation of both single and dual catalyst CMRs was performed. In Figure 11a, it can be observed that the CO yield increases in both CMRs as the temperature is increased and the GHSV is reduced. Nonetheless, the effect of the secondary catalyst is also observed by reducing the CO yield, especially at higher operating temperatures. For instance, at the lowest GHSV and 650 °C, the CO yield at the retentate is reduced from 9% on the CMR with one catalyst to 6.5% on the dual CMR, while at 450 °C, it is reduced from 0.2% down to <0.05%.

Additionally, Figure 11b shows the H_2_ recovery obtained by the CMRs at different space velocities and temperatures. In both CMRs, H_2_ recovery increases with higher temperatures and reduced GHSV, since these conditions are favorable for higher CH_4_ conversions. Additionally, it can clearly be seen that H_2_ recovery increases in the dual CMR particularly as the temperature increases. For instance, the operation of the dual bed at 600 °C is expected to produce more H_2_ than the conventional single-stage CMR. Additionally, lower CO yields intrinsically mean not only higher H_2_ generation and enriched CO_2_ streams at the retentate, but also the potential reduction of CO poisoning of the membrane. Several studies have shown that severe reductions in H_2_ permeance occur in the presence of CO mainly caused by the adsorption of CO on the Pd surface, hindering the active sites available for H_2_ to adsorb [40]. Reacting CO with H_2_O in the catalyst section allows the membrane to be less exposed to CO reducing poisoning. Furthermore, the presence of the WGS can potentially decrease coking when operating at low steam-to-carbon ratios, as carbon formation is thermodynamically favored by the dissociation of CO [41].

(23) $$2\mathrm{CO}\;↔C+{\mathrm{CO}}_{2}$$

**Figure 12:**
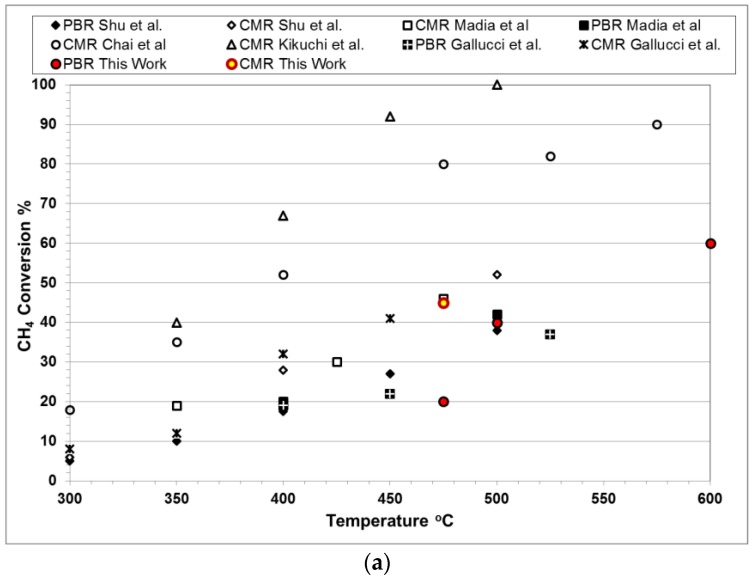

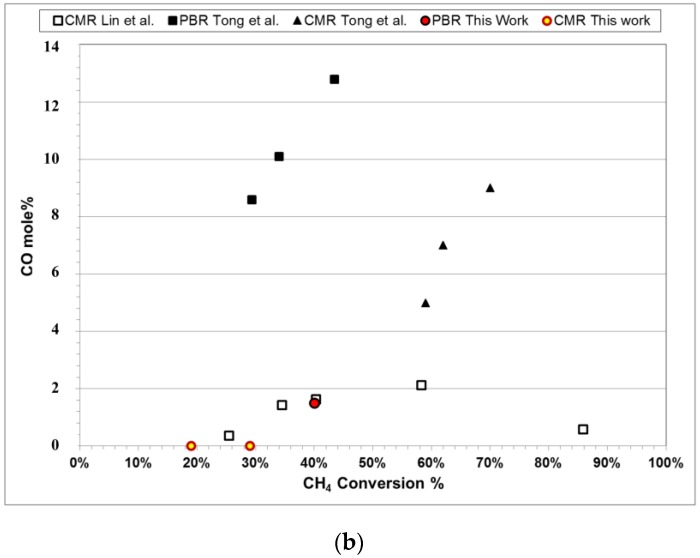
Comparison of the results presented in this work against those presented in the literature for (**a**) methane conversion and (**b**) CO yield at 450–500 °C and 2–5 bar [10,42,43,44].

The results obtained in the present work were compared against those shown in the literature for methane steam reforming, as shown in Figure 12a. The conversion of methane in traditional packed bed reactors (PBR) and membrane reactors (CMR) from different literature sources was plotted against different temperatures as reported by Gallucci et al. [10], and it is shown to be in agreement with previously-reported values. Furthermore, various CO mole fractions at the outlet of the reactor were graphically represented as a function of different methane conversions, as shown in Figure 12b. The composition of CO shown experimentally by this work is significantly lower than those shown in other sources, suggesting that the additional WGS catalyst in the CMR helped in decreasing the residual CO.

## 4. Conclusions

The concept of catalyst packing in series was explored through the development of a catalytic membrane reactor (CMR) module utilizing two catalysts positioned in series. In the process system under consideration, the methane steam reforming catalyst (MSR) is placed first to generate CO and H_2_, followed by a water-gas-shift layer placed in series used to react CO, thus producing a higher H_2_ yield. In particular, a tubular Pd/Au/Pd membrane was synthesized, characterized and accommodated throughout the reactor to remove the produced H_2_ in situ. The membrane was surrounded by a protective catalyst cage in order to protect the surface of the membrane, which helped in preventing carbon deposition on the surface of the membrane. The performance of this novel reactor was comparatively assessed against a conventional packed bed reactor (PBR) with no stages, as well as five-catalyst stages. In addition, a computational fluid dynamics (CFD) simulation framework in 2D was developed to further analyze the characteristics of the CMR. The experimental results for the conventional and CMR module are in agreement with the simulation-generated performance characterization ones. Moreover, the membrane used in this work displayed experimental H_2_ permeances of 70 and 80 Nm^3^·m^−2^·h^−1^·bar^−0.5^ at 350 and 450 °C, respectively. Notice that this configuration is reported for the first time in the pertinent literature and exhibited excellent technical performance. Indeed, it was demonstrated that excellent H_2_/He selectivity is attainable after catalyst activation at 600 °C while producing H_2_ with a purity of >99.9% over 350 h of continuous operation under MSR/WGS conditions and 300 h under pure H_2_ testing conditions. The cumulative testing time of the membrane was 650 h or one month.

The dual CMR was operated at a temperature of 475 °C, a pressure of 5 bar, steam-to-carbon ratios of three and five and gas hourly space velocities between 1000 and 6000 h^−1^. This dual CMR showed higher methane conversion than the conventional reactor. Please notice that this effect, also known to be critically related to key process intensification objectives, was more noticeable at low space velocities. The CMR module had a DaPe number ranging between 0.5 and 1, demonstrating the effective membrane performance at the specified conditions. Furthermore, the dual CMR module showed a significant reduction in the CO content, which was shown to be the result of the subsequent “packing step” with the WGS catalyst introduced in the proposed module design.

## Figures and Tables

**Table 1:** Palladium-based catalytic membrane reactors used for methane steam reforming (MSR) and water gas shift (WGS) reactions.

Membrane Type	Thickness (μm)	Membrane Area (cm^2^)	Reaction	Pressure (bar)	Steam/Carbon Ratio	Temperature (°C)	Reference
Pd/Ag	50	5.3	MSR	1.22	3–9	300–500	[10]
Pd/Ag/PSS *	10.3	10.7	MSR	1.36	3	400–550	[11]
Pd/PSS	20	60	MSR	9–20	3	400–500	[12]
Pd	4.5–22.5	6.3	MSR	1	3	500	[13]
Pd	4–5	175	MSR	<10	2–4	525	[7]
Pd/Ag	200	46	MSR	1–4	2–5	300–400	[14]
Pd/Ag	1000	18.5	MSR	6–10	2.9	500	[15]
Pd–Ru/YSZ **	5	13.28	MSR	35	3	580	[16]
Pd	20	25	WGS	3	1–5	400	[17]
Pd	1.4	21.5	WGS	2	3	350	[18]
Pd-Pd/Ag	7–10.3	50	WGS	1–12	1.1–2.6	350–450	[19]
Pd/Ag	25–40	15.7	WGS	1–4	7.4	200–300	[20]
Pd	10	200	WGS	7–20	2.5–3.5	420–440	[6]
Pd/Ag	2.2	6.8	WGS	26	5	400–450	[21]
Pd/Ag/alumina	4.5	17–35	WGS	2	NA	400	[22]

* PSS: porous stainless steel; ** YSZ: yttria-stabilized zirconia.

**Table 2:** Characteristics of the membrane at different phases of the synthesis.

Membrane Synthesis Step	Thickness/μm	He Leak (sccm/bar) at 25 °C
Initial support	NA	197,360
Oxidation and calcination	NA	91,830
Grading Pd(Al_2_O_3_)	2.8	66
Pd layer	6.9	3
Au deposition	0.2	1.39
Pd layer (final)	3.17	NA

**Table 3:** The Damkohler and Péclet (DaPe) number of the dual catalyst CMR at different space velocities at 5 bar and 475 °C.

GHSV (h^−1^)	DaPe Number
1170	0.47
1750	0.61
2270	0.56
2810	0.61
4680	0.77
6250	1.07

## Nomenclature

ρ	Density of the gas mixture	kg/m^3^
ε_p_	Porosity of the catalytic bed	–
β_F_	Forchheimer coefficient	kg/m^4^
τ	Tortuosity	–
d	Catalyst particle diameter	m
P_i_	Partial pressure of component *i* in the reaction zone	bar
${\mathrm{Pe}}_{m}$	Péclet number	–
Da	Damkohler number	–
r_i_	Reaction rate	kmol/kg_cat_/h
R_i_	Rate of generation and/or destruction of component *i*	kmol/kg_cat_/h
k_i_	Reaction rate constant	varies
K_i_	Equilibrium constant	varies
GHSV	Gas hourly space velocity	h^−1^
$N_{i}$	Flux of component *i*	mol/s
R_g_	Ideal gas constant	J/K/mol
$\bar{P_{H2}}$	Hydrogen permeance of the membrane	Nm^3^·m^−2^·h^−1^·bar^−0.5^
T	Temperature	K
Δ	Delta index	–
t	Membrane’s thickness	µm
u	Velocity field	m/s
F	External body forces (gravity, electric, etc.)	N
I	Identity matrix	–
*X*	Conversion of methane	–
$D_{AB}$	Binary diffusion coefficient between components A and B	cm^2^/s
$M_{A}$	Molecular weight of component A	g/mol
$\varphi_{D,AB}$	Collision integral for diffusion	Å
σ_AB_	Lennard–Jones potential between molecules A and B	–
